# Prescripción e indicaciones de uso de fluoroquinolonas en un grupo de pacientes ambulatorios de Colombia

**DOI:** 10.7705/biomedica.5103

**Published:** 2020-06-30

**Authors:** Manuel Enrique Machado-Duque, Katherine Mercado-Gómez, María Camila Bernal-Chica, Stephanie Uribe-Vélez, Jorge Enrique Machado-Alba

**Affiliations:** 1 Grupo de Investigación en Farmacoepidemiología y Farmacovigilancia, Universidad Tecnológica de Pereira - Audifarma, S. A., Pereira, Colombia Grupo de Investigación en Farmacoepidemiología y Farmacovigilancia Universidad Tecnológica de Pereira - Audifarma, S. A. Pereira Colombia; 2 Grupo de Investigación Biomedicina, Fundación Universitaria Autónoma de las Américas, Pereira, Colombia Fundación Universitaria Autónoma de las Américas Grupo de Investigación Biomedicina Fundación Universitaria Autónoma de las Américas Pereira Colombia

**Keywords:** fluoroquinolonas, usos terapéuticos, uso fuera de lo indicado, farmacoepidemiología, Fluoroquinolones, therapeutic uses, off-label use, pharmacoepidemiology

## Abstract

**Introducción.:**

Existen evidencias sobre el uso indiscriminado de antibióticos en el tratamiento de diversas enfermedades.

**Objetivo.:**

Determinar los patrones de prescripción y de indicaciones de uso de las fluoroquinolonas en un grupo de pacientes ambulatorios en Colombia.

**Materiales y métodos.:**

Se llevó a cabo un estudio descriptivo farmacoepidemiológico del tipo de prescripción e indicaciones de uso a partir de una base de datos poblacionales que incluía pacientes con prescripciones ambulatorias de fluoroquinolonas entre mayo y octubre de 2018. Se recabó la información sobre las variables sociodemográficas, farmacológicas y clínicas (diagnóstico según la Clasificación Internacional de Enfermedades, versión 10) y se estableció la proporción del uso de fluoroquinolonas en indicaciones aprobadas y no aprobadas por las agencias reguladoras.

**Resultados.:**

Se identificaron 23.373 pacientes que habían recibido fluoroquinolonas; su edad media era de 47,9 ± 18,1 años y 15.767 eran mujeres (67,5 %). La ciprofloxacina fue el medicamento más prescrito (n=19.328; 82,7 %), seguida de la norfloxacina (n=3.076; 13,2 %), la levofloxacina (n=573; 2,5 %) y la moxifloxacina (n=394; 1,7 %). Las principales indicaciones fueron la infección de las vías urinarias en sitio no especificado (n=10.777; 46,1 %), la diarrea y la gastroenteritis de presunto origen infeccioso (n=3.077, 13,2 %) y la cistitis aguda (n=956; 4,2 %). El 76 % (n=17.759) de las prescripciones correspondía a indicaciones aprobadas y el resto a usos no aprobados, como la rinofaringits o las infecciones de tejidos blandos. El ser hombre (*odds ratio*, OR=1,26; IC_95%_: 1,18-1,34) y tener menos de 35 años (OR=1,92; IC_95%_:1,48-1,50) se asociaron con una mayor probabilidad de uso de fluoroquinolonas en indicaciones no aprobadas.

**Conclusión.:**

Las fluoroquinolonas, en particular la ciprofloxacina, se están prescribiendo especialmente a mujeres con infecciones de las vías urinarias, pero hasta la cuarta parte de los pacientes las recibieron para usos no aprobados por las agencias reguladoras.

## Abstract (en)

**Introduction:**

There is evidence of the indiscriminate use of antibiotics for different pathologies.

**Objective:**

To determine the prescription patterns and indications for the use of fluoroquinolones in a group of outpatients in Colombia.

**Materials and methods:**

We conducted a descriptive pharmaco-epidemiological study on prescription-indication using a population database where patients with outpatient fluoroquinolone prescriptions were included from May to October, 2018. We obtained the information on sociodemographic, pharmacological, and clinical variables, as well as on the diagnosis according to the International Classification of Diseases, version 10, and we established if the use was approved by the regulatory agencies or if it was off-label.

**Results:**

A total of 23,373 patients were identified who were using fluoroquinolones; their mean age was 47.9 ± 18.1 years and women predominated (n=15,767, 67.5%). Ciprofloxacin was the medication most commonly prescribed (n=19,328, 82.7%), followed by norfloxacin (n=3076, 13.2%), levofloxacin (n=573, 2.5%), and moxifloxacin (n=394; 1.7%). The main indications were urinary tract infection in unspecified site (n=10,777, 46.1%), diarrhea and gastroenteritis of presumed infectious origin (n=3077, 13.2%), and acute cystitis (n=956; 4.2%). The prescriptions followed approved indications in 76% (n=17,759) of cases while the rest were used off-label or without indication for nasopharyngitis or soft-tissue infections, for example. Being male (OR=1.26, 95%CI:1.18-1.34) and under 35 years of age (OR=1.92, 95%CI:1.48-1.50) were associated with a greater probability of using fluoroquinolones in unapproved indications.

**Conclusions:**

Fluoroquinolones, particularly ciprofloxacin, are being prescribed especially to women with urinary tract infections, but up to a quarter of the patients received them for unapproved indications by regulatory agencies.

En los últimos años se ha evidenciado el uso indiscriminado de antibióticos en el tratamiento de diversas enfermedades ^1^, lo que contradice las disposiciones de la Organización Mundial de la Salud (OMS) sobre el uso racional de medicamentos ^2^. En un estudio del 2018 se encontró que las fluoroquinolonas se prescribían para tratar infecciones para las que no estaban indicadas en el 5,1 % de los casos y en el 19,9 % de las prescripciones ambulatorias para casos en los que no eran la primera línea de tratamiento ^3^. Por otra parte, se ha reportado que solo el 52 % de las formulaciones era adecuado para el tratamiento de primera línea ^4^, lo que podría asociarse con el incremento de la resistencia de agentes patógenos comunes como *Escherichia coli* en las infecciones urinarias ^5^^,^^6^ en hasta el 50 % de los casos en algunas regiones ^7^. Por último, cabe resaltar la importancia de su uso en el tratamiento de rescate de pacientes que presentan efectos secundarios o resistencia a los antibióticos de primera línea en infecciones de interés en salud pública como la tuberculosis, ya que se ha registrado una alta tasa de éxito e, incluso, reducción de la mortalidad ^8^.

En el documento *Critically Important Antimicrobials for Human Medicine* de la OMS, elaborado por expertos en antimicrobianos para la salud humana, se plantea que la gestión del riesgo de resistencia a los antibióticos debería priorizarse en algunos grupos de antibióticos (fluoroquinolonas y cefalosporinas de tercera y cuarta generación) ^9^. Además, en el plan de acción mundial contra la resistencia a los antimicrobianos se cita una disminución del uso general de antibióticos, con excepción de las fluoroquinolonas, especialmente en el ámbito comunitario ^10^. En los Estados Unidos se ha estimado que 115 de las recetas de cada 1.000 casos corresponden a fluoroquinolonas, siendo este el tercer grupo de antibióticos más prescrito en la población adulta ^11^.

En los estudios de Colombia, se ha establecido que las fluoroquinolonas están entre los grupos de antibióticos más formulados después de las penicilinas, las cefalosporinas y las sulfonamidas, aunque con grandes diferencias entre ciudades: desde 14,2 hasta 61,1 prescripciones por cada 1.000 pacientes; sin embargo, no se han publicado estudios sobre las indicaciones y la forma de utilización de este grupo de antimicrobianos en el país ^12^^,^^13^.

El sistema de salud de Colombia ofrece cobertura universal en uno de dos regímenes de afiliación, uno contributivo, que es pagado por el trabajador y el empleador, y el otro subsidiado, pagado por el Estado, que incluye algunas de las fluoroquinolonas para uso ambulatorio y hospitalario.

Debido a su frecuente uso, al riesgo de utilización inapropiada e, incluso, a los potenciales problemas de seguridad, el objetivo de este estudio fue determinar los patrones de utilización, las indicaciones y los usos de las fluoroquinolonas en un grupo de pacientes ambulatorios de Colombia durante el 2018.

## Materiales y métodos

Se llevó a cabo un estudio retrospectivo observacional para establecer la prescripción y las indicaciones de las formulaciones de fluoroquinolonas en pacientes ambulatorios mayores de 18 años de edad y de cualquier sexo afiliados a alguna de las seis entidades promotoras de salud (EPS) a las cuales Audifarma, S.A. empresa encargada del proceso logístico de dispensación de medicamentos a instituciones dispensó medicamentos entre mayo y octubre del 2018. La información se obtuvo de la base de datos poblacionales de dispensación de Audifarma, S.A., la cual ha sido validada y utilizada en diferentes estudios farmacoepidemiológicos. Se excluyeron los pacientes sin diagnóstico de infección y se incluyeron todos aquellos con infecciones listadas en la Clasificación Internacional de las Enfermedades, versión 10 (CIE-10: A00-A99, B00-B99).

Se elaboró una base de datos con los registros de todos los pacientes a quienes se dispensaron fluoroquinolonas y con las variables de interés, información que fue validada por el equipo de investigación (revisión de valores faltantes, identificación y validación de datos extremos y unificación de valores).

Se consideraron los siguientes grupos de variables:

1. Sociodemográficas: edad, sexo, ciudad de residencia y EPS.
2. Farmacológicas: nombre y código según el *Anatomical Therapeutic Chemical Classification System* (ATC) de las fluoroquinolonas prescritas, dosis, intervalo y duración de la terapia. Se calcularon las dosis diarias definidas como forma de comparación del uso del medicamento.
3. Clínicas: diagnóstico principal asociado con cada prescripción de fluoroquinolonas según el código CIE-10, y la indicación aprobada o no aprobada según los registros de la *Food and Drug Administration* (FDA) de los Estados Unidos y el Instituto Nacional de Vigilancia de Medicamentos y Alimentos (Invima) de Colombia. Se categorizaron como indicaciones aprobadas para la ciprofloxacina el ántrax, la infección abdominal adquirida en la comunidad, la osteomielitis, la infección por *Yersinia pestis*, la neumonía por *Pseudomonas aeruginosa*, la prostatitis, la infección gastrointestinal, el uso como medicamento alternativo en la artritis séptica y la infección de vías urinarias.Las indicaciones aprobadas para la norfloxacina incluyeron la prostatitis, la infección urinaria, la gonorrea no complicada, la peritonitis bacteriana espontánea y la diarrea del viajero. Las indicaciones para la levofloxacina fueron el ántrax, la exacerbación aguda de la enfermedad pulmonar obstructiva crónica (EPOC) (sospecha de *P. aeruginosa)*, la erradicación de *Helicobacter pylori*, la neumonía adquirida en la comunidad, la infección por *Y. pestis,* la prostatitis, la rinosinusitis bacteriana aguda, la celulitis purulenta con sospecha de infección por bacilos Gram negativos, la tuberculosis y la infección urinaria. El uso de la moxifloxacina se contempló para la bronquitis crónica con exacerbación aguda, la neumonía adquirida en la comunidad, las infecciones abdominales, la rinosinusitis bacteriana aguda, la celulitis purulenta con sospecha de infección por bacilos Gram negativos y la tuberculosis.
4. Comedicaciones y comorbilidades: se completaron los registros con las dispensaciones concomitantes que recibían los pacientes utilizando estas como variables sustitutas de la siguiente manera: a) antihipertensivos (hipertensión arterial); b) hipolipemiantes (dislipidemia, riesgo cardiovascular); c) aspirina, antiagregantes plaquetarios (enfermedad coronaria); d) antidepresivos (depresión, ansiedad); e) antidiabéticos e insulinas (diabetes mellitus); f) antirreumáticos modificadores de enfermedad (artritis reumatoide); g) antiinflamatorios no esteroideos (AINE) (inflamación o dolor), y h) analgésicos (dolor).

El estudio recibió el aval del Comité de Bioética de la Universidad Tecnológica de Pereira como investigación sin riesgo. Se respetaron los principios establecidos por la Declaración de Helsinki. En ningún caso se emplearon datos personales de los pacientes.

### Plan de análisis

La base de datos se analizó con el programa SPSS^™^, versión 25.0, para Windows (IBM, USA). Las variables cuantitativas se resumieron en medidas de tendencia central y de dispersión, y las categóricas, en frecuencias y proporciones.

Se hicieron análisis bivariados mediante la prueba de ji al cuadrado en busca de posibles variables asociadas con el uso de fluoroquinolonas en indicaciones no aprobadas por las agencias reguladoras.

Se desarrolló un modelo multivariado de regresión logística binaria que incluyó las variables asociadas en los análisis bivariados, así como aquellas con suficiente plausibilidad o asociación reportada para identificar las que se pudieran asociar con el uso de fluoroquinolonas en indicaciones no aprobadas después del ajuste. Las diferencias significativas se establecieron con un valor de p menor de 0,05.

## Resultados

Se identificaron 23.373 personas con prescripción de alguna fluoroquinolona; su edad media fue de 47,9 ± 18,1 años, y la mayoría eran mujeres (n=15.767, 67,5 %). La ciudad con el mayor número de pacientes fue Bogotá (n=7.152; 30,6 %), seguida de Barranquilla (n=1.962; 8,4 %), Cali (n=1.902; 8,1 %), Cartagena (n=1.515; 6,5 %), Pereira (n=1.024; 4,4 %), Santa Marta (n=966; 4,1 %), Bucaramanga (n=791; 3,4 %) y Medellín (n=625; 2,7 %). Los restantes pacientes eran atendidos en otras 88 ciudades.

La mayoría de las prescripciones de fluoroquinolonas habían sido formuladas por medicina general (n=21.332; 91,3 %), seguidas de cirugía general (n=566; 2,4 %), urología (n=235; 1,0 %), gastroenterología (n=178; 0,8 %), odontología (n=194; 0,8 %), y medicina interna (n=139; 0,6 %), en tanto que las prescripciones de los restantes 729 pacientes (3,1 %) habían sido recetadas por otras 27 especialidades. Se encontraron 25 prescripciones hechas por personal no autorizado por las normas como personal de enfermería, nutrición, optometría y terapia respiratoria.

La ciprofloxacina fue la fluoroquinolona más prescrita, seguida de la norfloxacina, en tanto que la levofloxacina y la moxifloxacina solo se formularon en el 4,1 % de los casos. En el cuadro 1 se presentan los patrones de prescripción con frecuencias de uso, dosis, relación con las dosis diarias definidas y la distribución por sexo.

**Cuadro 1:** Patrones de prescripción, frecuencias de uso, dosis, relación con la dosis diaria definida y distribución por sexo en pacientes ambulatorios de Colombia con prescripción de fluoroquinolonas, 2018

	Dosis prescrita (mg/día)					Sexo		Edad	
Medicamento	(n)	(%)	Media	Moda	nDDD^a^	M (%)	F (%)	(media ± DE)
Ciprofloxacina	19.328	82,7	1.044	1.000	1,04	33,7	66,3	47,5 ± 18,8
Norfloxacina	3.076	13,2	789	800	0,98	24,3	75,7	49,2 ± 18,1
Levofloxacina	573	2,5	777	500	1,55	36,3	63,7	54,7 ± 16,0
Moxifloxacino	394	1,7	616	400	1,54	35,0	65,0	49,3 ± 17,3

DDD: dosis diaria definida; DE: desviación estándar M: masculino; F: femenino

^a^ Relación entre la dosis media y la dosis diaria definida

Las principales indicaciones encontradas fueron la infección de las vías urinarias de sitio no especificado (n=10.777; 46,1 %), la diarrea y la gastroenteritis de presunto origen infeccioso (n=3.077; 13,2 %), la cistitis aguda (n=956; 4,2 %), la infección de tejidos blandos (n=810; 3,3 %), la vaginitis, la vulvitis y la vulvovaginitis (n=742; 3,1 %), las infecciones de etiología viral (n=568; 2,3 %), y las de etiología fúngica (n=81; 0,1 %). Además, se hallaron 10 pacientes en estado de gestación con prescripción de fluoroquinolonas.

El 76 % (n=17.759) de las prescripciones respondía a indicaciones aprobadas por la FDA, en tanto que el 24 % (n=5.614) restante se usaba sin el respaldo científico de ensayos clínicos controlados o guías de práctica clínica. En el cuadro 2 se presentan los principales diagnósticos no aprobados para su uso.

**Cuadro 2:** Principales diagnósticos no aprobados registrados en la prescripción de fluoroquinolonas a pacientes ambulatorios de Colombia, 2018 (n=23.373)

Diagnóstico	n	(%)
Rinofaringitis	943	4,0
Infección de tejidos blandos	810	3,5
Vaginitis, vulvitis y vulvovaginitis	757	3,2
Gastritis	708	3,0
Otitis	633	2,7
Conjutivitis	110	0,5
Infección de etiología fúngica	81	0,3
Otros diagnósticos no aprobados	1.572	6,7

El 34,2 % (n=8.003) de los pacientes recibía alguna otra medicación, 21,8 % de ellos con dos o más fármacos, siendo los más frecuentemente prescritos los antihipertensivos (n=5.346; 22,9 %), especialmente los bloqueadores del receptor de angiotensina (n=3.305; 14,1 %), seguido de los beta-bloqueadores (n=1.632; 7,0 %), los bloqueadores de los canales de calcio (n=1.606; 6,9 %), la hidroclorotiacida (n=1.265; 5,4 %), los inhibidores de la enzima convertidora de angiotensina (n=897; 3,8 %), la furosemida (n=649; 2,8 %) y la espironolactona (n=290; 1,2 %). También se encontraron comedicaciones frecuentes con las estatinas (n=3.940; 16,9 %), los fibratos (n=520; 2,2 %), los antidepresivos (n=2.082; 8,9 %), los antirreumáticos modificadores de enfermedad (n=373; 1,5 %), o antidiabéticos como la metformina (n= 1.180; 5,0 %), las insulinas (n=533; 2,3 %), y las incretinas (n=329; 1,4 %).

El análisis multivariado evidenció que el pertenecer al grupo de menores de 35 años, o de 35 a 65 años, ser hombre, ser tratado en Bogotá o Barranquilla y recibir levofloxacina o insulina se asociaron con una mayor probabilidad de que la fluoroquinolona fuera prescrita para indicaciones no aprobadas por la FDA, en tanto ser tratado en Santa Marta, Medellín o Bucaramanga, recibir tratamiento con bloqueadores alfa-adrenérgicos o con fibratos se asociaron con una menor probabilidad de uso no aprobado (cuadro 3)

**Cuadro 3:** Variables asociadas con usos no aprobados de fluoroquinolonas mediante una regresión logística binaria en pacientes ambulatorios de Colombia, 2018

Variable	Significación	Odds ratio	Intervalo de confianza del 95 %	
	estadística		Inferior	Superior
Edad menor de 35 años	<0,001	1,928	1,483	2,507
Edad entre 35 y 65 años	0,001	1,546	1,196	1,998
Edad entre 65 y 85 años	0,238	1,17	0,902	1,518
Edad mayor de 85 años	<0,001	Ref	Ref	Ref
Ser hombre	<0,001	1,262	1,183	1,347
Ser tratado en Bogotá	<0,001	1,189	1,104	1,28
Ser tratado en Barranquilla	0,001	1,189	1,072	1,319
Ser tratado en Santa Marta	<0,001	0,696	0,59	0,82
Ser tratado en Medellín	<0,001	0,719	0,603	0,858
Ser tratado en Bucaramanga	0,002	0,768	0,647	0,911
Utilizar levofloxacina	<0,001	8,995	7,465	10,837
Utilizar alfa-bloqueadores	0,002	0,621	0,463	0,834
Utilizar fibratos	0,038	0,783	0,621	0,987
Utilizar insulinas	<0,001	1,6	1,301	1,968

### Discusión

Con el presente estudio se lograron determinar las indicaciones de uso de las fluoroquinolonas en pacientes ambulatorios en una muestra representativa de pacientes mayores de 18 años en Colombia, por lo que constituye un punto de partida para indagar la forma en que se están empleando las fluoroquinolonas, y sería útil en la elaboración de estrategias de racionalización y mejoramiento de su empleo. Esto reviste un especial interés porque se trata de medicamentos ampliamente utilizados en la práctica clínica, que presentan una creciente resistencia frente a diversos microorganismos y han suscitado nuevas alertas de seguridad ^7^^,^^14^^,^^15^.

Uno de los resultados importantes fue que el uso ajustado a las recomendaciones aprobadas se registró en cerca del 75 % de los casos, lo que se explicaría por la elevada frecuencia de su uso en infecciones urinarias para las cuales están aprobadas como primera (pielonefritis aguda) o segunda línea (cistitis aguda) de tratamiento ^16^^,^^17^. Para otras indicaciones, como las infecciones de tejidos blandos, cuyo tratamiento con fluoroquinolonas fue aprobado por las agencias reguladoras solo en algunos casos, existiría cierta controversia, puesto que ninguna guía de práctica clínica las recomienda como elección o alternativa, por lo que su uso se puede considerar inapropiado por estar asociado a fracasos terapéuticos, a la posible adquisición de resistencia bacteriana y a la aparición de efectos secundarios evitables ^18^. Además, merece especial consideración el hallazgo de su formulación para infecciones de etiología viral o fúngica en las cuales su uso no está respaldado por ningún estudio ^19^^,^^20^, o su prescripción en mujeres gestantes en quienes su empleo se restringe a los casos de ántrax o a situaciones en las que el beneficio pudiera superar los riesgos ^21^.

En otros estudios, como el llevado a cabo en los Estados Unidos por Almalki, *et al.*, se encontró que cerca del 53 % de las prescripciones de fluoroquinolonas se hacía para indicaciones no aprobadas, lo cual contrasta con el 76 % de usos aprobados hallado en el presente trabajo ^22^. En el estudio de Ray, *et al.*, se reportó que en los casos de uso de antimicrobianos en general, el 25 % se había prescrito de manera inapropiada y el 18 %, sin indicación documentada; en cuanto a las fluoroquinolonas, la situación empeoraba debido a que en el 38 % de los casos su uso había sido inapropiado y en el 20 % se había prescrito sin indicación documentada ^23^. Por otra parte, en un estudio publicado por Suzuki, *et al*., sobre la prescripción de fluoroquinolonas en el momento del alta hospitalaria, se halló que en el 41,1 % de los casos su uso había sido inapropiado, y la frecuencia del mal uso aumentaba en aquellos centros hospitalarios sin estrategias de uso racional de antimicrobianos ^24^.

Para contrarrestar las prescripciones que no se ajustan a los usos aprobados deben implementarse intervenciones de educación continua, así como la difusión de guías de práctica clínica para mejorar el uso de los medicamentos.

Como era de esperarse, en este estudio la mayoría de estas prescripciones estuvo a cargo de médicos generales, lo que responde a las características del sistema de salud del país, en el que predomina la atención de primer nivel, y se sabe que las fluoroquinolonas están indicadas principalmente para el manejo de infecciones leves y moderadas con un nivel de atención de baja complejidad. Llama la atención el hallazgo de prescripciones hechas por personal de otras áreas de la salud, cuando la ley establece que deben ser expedidas por los profesionales competentes ^25^. Incluso se ha podido establecer que los estudiantes de medicina prescriben medicamentos ^26^ y que se despachan antibióticos en las farmacias sin fórmula médica ^27^^,^^28^. Esta situación debe controlarse debido al incremento de la resistencia antibiótica y la necesidad de aplicar la racionalidad en la prescripción.

En este estudio, la ciprofloxacina y la norfloxacina, las fluoroquinolonas más prescritas, se habían formulado en las dosis recomendadas por la OMS y en los intervalos adecuados, en tanto que las dosis de levofloxacina y moxifloxacina excedían en 50 % la dosis diaria definida (aunque tales cantidades siguen estando dentro del rango recomendado para la mayoría de indicaciones, en especial las infecciones de las vías urinarias y de las respiratorias inferiores), y en los intervalos adecuados ^16^^,^^29^.

Al igual que en un estudio realizado en los Estados Unidos, se halló que los hombres tenían mayores probabilidades de recibir las fluoroquinolonas en indicaciones no aprobadas (OR=3,26 *Vs.* 1,26 en este estudio), pero en aquel se encontró que los adultos mayores de 80 años también tenían ese riesgo, lo cual difiere del presente estudio en el que tal asociación se estableció para los menores de 65 años ^(22)^. En cuanto al uso de bloqueadores alfa-adrenérgicos, en este estudio se encontró que era un factor protector frente a las prescripciones no aprobadas, probablemente debido a su estrecha relación con el tratamiento de la hipertrofia prostática benigna y complicaciones como la prostatitis o las infecciones urinarias en hombres, para las cuales el uso de fluoroquinolonas está aprobado, así como lo está el uso de antimicrobianos conjuntamente con los bloqueadores alfa por haberse demostrado que logran una mejoría significativa según la escala *National Institutes of Health Chronic Prostatitis Symptom Index* (NIH-CPSI) ^30^.

Se han planteado posibles explicaciones para el uso de las fluoroquinolonas en indicaciones no aprobadas: en algunos estudios se ha encontrado que la prescripción inapropiada de antibióticos es bastante frecuente para las infecciones de las vías respiratorias superiores, especialmente por parte de los médicos de atención primaria, y que los pacientes desconocen el origen de la sintomatología y la naturaleza autolimitada de estas afecciones ^(23,31)^. En respuesta, se ha encontrado que estrategias como los programas de optimización del uso de los antimicrobianos (*antimicrobial stewardship*) podrían evitar las prescripciones inapropiadas de fluoroquinolonas ^24^^,^^31^.

El presente estudio tuvo algunas limitaciones: solo se tenía información sobre el diagnóstico principal asociado con la prescripción de las fluoroquinolonas cuando su uso podría ser el indicado para el diagnóstico secundario o para otro no registrado; además, se desconocían las potenciales contraindicaciones, las reacciones adversas o el nivel de cumplimiento del tratamiento. Por otra parte, la distribución por ciudades dependió de los lugares donde cada EPS tenía afiliados. Sin embargo, el estudio fue riguroso en la obtención de la información y el tamaño de la muestra en los diversos lugares del país, pues se contaba con una base de datos confiable utilizada ampliamente en estudios farmacoepidemiológicos previos, así como con los registros de dispensación de cada medicamento, de los diagnósticos y las variables sociodemográficas.

Los hallazgos permiten concluir que la mayoría de los pacientes que estaban recibiendo fluoroquinolonas, especialmente ciprofloxacina y norfloxacina, eran en su mayoría mujeres que estaban siendo tratadas principalmente para infecciones del tracto urinario para las cuales su uso está aprobado por la FDA y el Invima. Sin embargo, en una cuarta parte de los casos se utilizaron para indicaciones no aprobadas, especialmente la levofloxacina en hombres y en menores de 35 años. Por ello se recomienda hacer nuevos estudios que profundicen en las formas de uso, las indicaciones y las reacciones adversas asociadas con las fluoroquinolonas en pacientes colombianos.
